# Delayed Tracheal Injury Following a Total Thyroidectomy Presenting as Anterior Neck Swelling: A Case Report

**DOI:** 10.7759/cureus.107512

**Published:** 2026-04-21

**Authors:** Nona Shalisha Sallay, H L D S Ariyaratne

**Affiliations:** 1 General Surgery, Colombo South Teaching Hospital, Kalubowila, LKA

**Keywords:** conservative management, delayed tracheal rupture, thyroidectomy, tracheal injury, tracheal mucosal herniation

## Abstract

Tracheal injury following thyroidectomy is rare, with delayed presentation being even less common. Tracheal mucosal herniation through a small tracheal wall defect represents an exceptionally unusual postoperative complication. This report describes a 49-year-old woman who developed a delayed anterolateral tracheal defect with mucosal herniation two weeks after a total thyroidectomy. Conservative management led to complete recovery. This case highlights the importance of maintaining a high index of clinical suspicion for airway complications in the late postoperative period and demonstrates that small, contained tracheal defects can be safely managed nonoperatively.

## Introduction

Surgical removal of the entire thyroid gland is often used to treat both benign and malignant thyroid problems. When performed by skilled surgeons, the likelihood of complications is low, affecting only about 3-5% of patients. Postoperative complications following a total thyroidectomy may include recurrent laryngeal nerve injury resulting in dysphonia, hypocalcaemia secondary to parathyroid gland dysfunction or devascularization, postoperative haematoma, and surgical site infection [1,2]. Although it is rare, occurring in fewer than 1% of cases, damage to the trachea can happen during thyroid surgery [2]. If the trachea is accidentally perforated or torn during the operation, it is typically identified right away and promptly repaired, which generally prevents serious complications, such as life-threatening airway obstruction, respiratory compromise, mediastinitis, pneumothorax, and tracheocutaneous fistula formation [1,2].

However, delayed tracheal rupture following thyroidectomy is extremely rare but potentially life-threatening. It may arise from unrecognized intraoperative trauma or be secondary to tracheal wall necrosis. Symptoms typically present within one to two weeks after the operation [1-3]. Tracheal injuries related to trauma, bronchoscopy, or oncologic resection have been widely described. By contrast, the literature on inadvertent tracheal perforation specifically associated with thyroid surgery is scarce. Some reports note the lack of published guidelines addressing treatment strategies for such cases [2,4]. Proposed mechanisms include excessive dissection or peeling of the tracheal wall during difficult thyroidectomy, oncologic involvement requiring aggressive mobilization, or necrosis of adherent pathological tissues [3]. A diagnostic evaluation often uses a combination of clinical evaluation, imaging (such as X-rays or CT scans), and bronchoscopy [3,5]. Therapeutic approaches vary, ranging from conservative management to more invasive interventions, such as segmental tracheal resection or reconstruction with muscular or musculocutaneous flaps when a standard repair is not feasible [3]. Given the rarity of this complication and the absence of standardized protocols, each new case provides valuable insights into its identification, evaluation, and management. This report details a patient who developed delayed tracheal injury 14 days after a total thyroidectomy, emphasizing the key aspects of diagnosis and management.

## Case presentation

**Figure 1:**
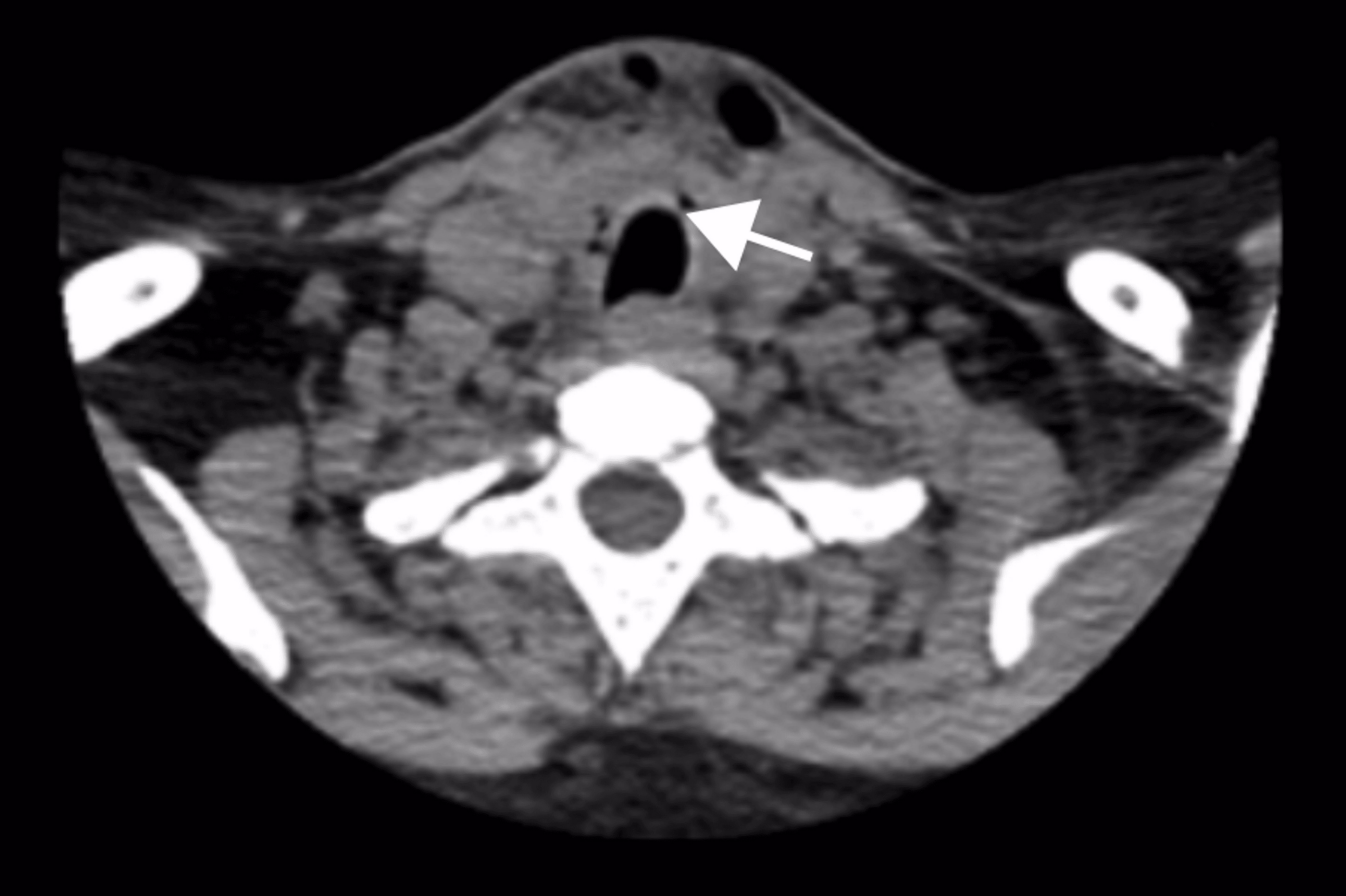
Axial noncontrast-enhanced computed tomography (NCCT) of the cervical spine showing an anterolateral tracheal defect. Axial view demonstrating a 1.5 mm defect (white arrow) in the anterolateral tracheal wall at the C7/T1 level.

A 49-year-old woman presented to the surgical clinic with a one-year history of gradually increasing neck swelling for which she had not sought previous medical attention. She was clinically and biochemically euthyroid. Examination revealed a clinically large multinodular goiter with mild rightward tracheal deviation. Neck ultrasonography confirmed multinodular changes. The left thyroid lobe measured 22 × 26 × 47 mm, and a suspicious right-sided thyroid nodule measuring 7 × 7 mm was identified, classified as TI-RADS (Thyroid Imaging Reporting and Data System) 5. Fine-needle aspiration cytology was reported as Bethesda V, suspicious for papillary thyroid carcinoma. Thyroid hormone levels remained within the normal range. She underwent an uncomplicated total thyroidectomy. Her immediate postoperative recovery was uneventful, with no respiratory distress, no voice changes, no hypocalcemia, and a normally healing surgical wound. She was discharged on the second postoperative day. Two weeks later, the patient presented with a dry cough consistently triggered by phonation, accompanied by soft neck swelling that demonstrated a cough impulse over the anterior neck. Despite these symptoms, she remained hemodynamically stable with an oxygen saturation of 99% in room air and normal bilateral air entry. A non-contrast CT scan of the neck demonstrated a 1.5 mm defect in the anterolateral tracheal wall at the level of C7/T1 (Figure 1).

**Figure 2:**
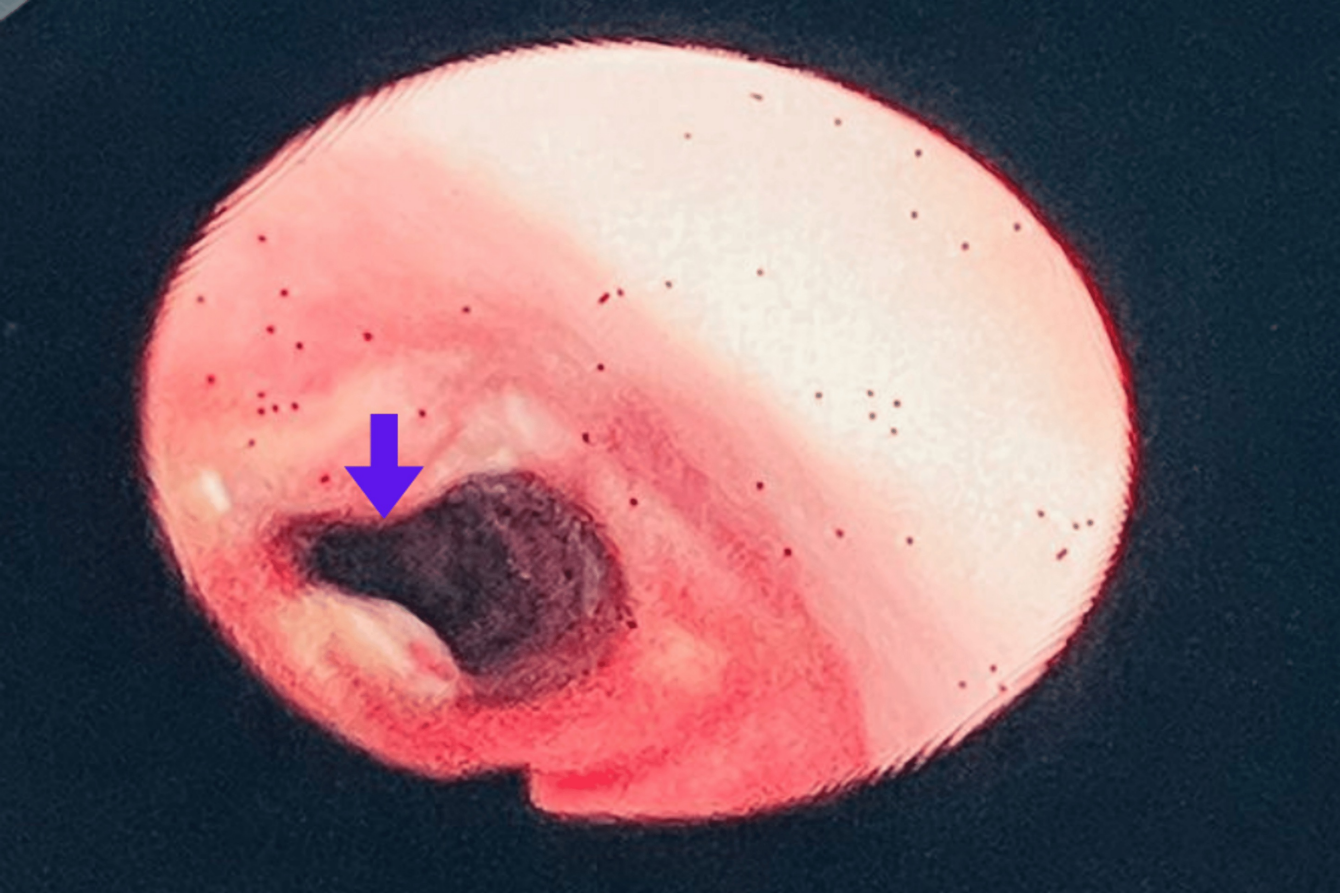
Fiber-optic laryngoscopy findings Fiber-optic laryngoscopy view of the larynx during phonation and inspiration. The image demonstrates no evidence of intraluminal tracheal collapse or dysfunction, despite the presence of mild tracheal narrowing. The violet arrow indicates the site of the anterior wall defect.

Air was tracking along this defect and communicating with an approximately 15 mL anterior air locule within the strap muscles. Mild subcutaneous edema was present, but no hematoma or fluid collections were identified. The flexible endoscopic laryngoscopy performed by the ENT team was normal, with no evidence of intraluminal collapse or vocal cord dysfunction (Figure 2).

Given the very small size of the defect, the absence of mediastinal extension, the contained nature of the mucosal herniation, and the patient's clinical stability, both the ENT and surgical teams agreed on a conservative approach. The patient was started on montelukast 10 mg nightly, cetirizine 10 mg nightly, nasal saline douching, fluticasone nasal spray, and steam inhalation as needed. She showed rapid symptomatic improvement on the third day, with complete resolution of the cough and reduction of neck swelling. No surgical intervention was required. At the two-week outpatient follow-up, sustained clinical improvement was noted, and she remains under ongoing routine follow-up for postoperative surveillance and thyroxine replacement therapy.

## Discussion

Incidence and risk factors for tracheal injury

Tracheal injury following a thyroidectomy is an uncommon yet potentially fatal complication. The incidence is estimated at around 0.06%, as demonstrated in a large series of 11,917 thyroidectomies reported by Gosnell et al. [4]. However, several authors caution that the true incidence is likely underreported due to missed diagnoses or delayed presentations [2,5-9]. Tracheal injury can occur intraoperatively as an accidental perforation or later present as tracheal necrosis [6-9].

In comparison, our patient underwent an initially uncomplicated total thyroidectomy and remained asymptomatic in the immediate postoperative period, with delayed symptoms only appearing two weeks later. This emphasizes that clinically occult injuries may present after an apparently uneventful recovery.

Risk Factors and Mechanisms

Multiple risk factors have been identified. Female sex, thyrotoxic goiter, long-standing multinodular goiter, and large compressive goiters can predispose patients to the weakening of the tracheal wall through repeated cycles of hyperplasia, degeneration, and fibrosis [1-3,6-9]. This fibrosis can obscure normal tissue planes, making dissection difficult and increasing the risk to the trachea, especially near Berry's ligament [4]. Prolonged intubation, elevated endotracheal tube cuff pressures, and trauma to the tube tip have also been implicated, as they can exceed mucosal perfusion pressure and cause pressure necrosis [10]. Sudden increases in intratracheal pressure caused by coughing, sneezing, retching, or shouting may precipitate rupture of already weakened tissue [6-9].

In comparison, our patient was a woman with a clinically large multinodular goitre, placing her within a recognized higher-risk group. Her cough-provoked symptoms support the possibility that a minor occult thermal or ischaemic injury became clinically evident after increased airway pressure.

Technical considerations and prevention

Intraoperative factors, such as excessive or careless diathermy use, especially in vascular thyroids, increase the risk of thermal devascularization of the tracheal wall [1-2,6-10]. Ligation of the inferior thyroid artery branches can reduce segmental tracheal blood flow, leading to ischemia and delayed necrosis [6]. Lugol iodine solution used preoperatively has been shown to reduce gland vascularity. In turn, it may decrease the amount of cautery needed during surgery [1-2,6]. Careful preservation of the tracheal blood supply, meticulous capsular dissection near Berry’s ligament, and minimization of energy device use close to the airway are recommended preventive strategies. Postoperatively, patients should be advised to avoid forceful coughing or activities that markedly increase intratracheal pressure during early healing [6-9]. Unlike cases with obvious intraoperative perforation, no tracheal breach was recognized during surgery in our patient, supporting the possibility of a small occult thermal or ischemic injury presenting later.

Detection and Intraoperative Repair

Acute perforations most often affect the posterolateral trachea near the Berry ligament, although anterior and lateral perforations have also been described [1]. When recognized intraoperatively, small perforations can usually be repaired with absorbable sutures and reinforced with a rotated strap muscle flap [1,4]. To identify small perforations, clinicians may fill the wound with saline and observe for air leaks (the Valsalva maneuver), a technique widely recommended since visual inspection alone often misses subtle defects [1,4]. By contrast, our patient had an initially uneventful postoperative recovery and only became symptomatic two weeks later, representing a delayed presentation rather than an immediately recognized intraoperative injury. This underlines the importance of preventive surgical technique even when no immediate defect is apparent.

Management of delayed presentation

Delayed perforations or necrosis usually present within two weeks after thyroidectomy [1,5]. Symptoms may include wound infection, subcutaneous emphysema, respiratory distress, hoarseness, neck swelling, hemoptysis, or retrosternal pain, often following an episode of vigorous coughing [1]. Imaging with neck and chest radiography or CT is essential to evaluate for pneumomediastinum or a tracheal injury site, while flexible bronchoscopy may help locate the defect [1,6]. Similar to prior reports, our patient presented within this recognized timeframe; however, rather than severe emphysema or respiratory compromise, she had subtle symptoms of a phonation-triggered cough and soft neck swelling while remaining clinically stable.

Surgical Versus Conservative Management

Management should be individualized according to airway stability, defect size, tissue viability, infection, and extent of emphysema. Stable patients with small contained defects and no respiratory distress may be managed conservatively with close observation, antibiotics when indicated, cough suppression, drainage of localized collections, compressive dressings, and serial reassessment [6-9].

Patients with progressive emphysema, sepsis, respiratory compromise, necrotic tissue, or larger defects usually require prompt operative intervention. Surgical options include debridement with primary closure, reinforcement using strap muscles or sternocleidomastoid flaps, temporary tracheostomy, vacuum-assisted wound therapy, or segmental tracheal resection with end-to-end anastomosis in extensive injuries [3,6-10]. Complex cases may require multidisciplinary management involving endocrine surgery, thoracic surgery, otolaryngology, anesthesia, and critical care teams [6,8].

In comparison, our patient remained hemodynamically stable, had no respiratory distress, and was found to have an exceptionally small anterolateral defect measuring 1.5 mm without surrounding necrosis or infection. These favorable features supported a non-operative approach with close surveillance, and she achieved an excellent outcome. This contrasts with many previously reported delayed ruptures that required aggressive surgical repair.

Clinical relevance of the present case

Most published delayed tracheal injuries involve larger defects, necrosis, severe emphysema, or airway instability requiring intervention [1,3,6-10].

In comparison, this case demonstrates that very small, localized delayed tracheal defects may present only with subtle symptoms and can be successfully managed conservatively when patients are carefully selected and closely monitored. It also reinforces the importance of maintaining suspicion for delayed airway injury even after an initially uncomplicated thyroidectomy.

## Conclusions

Delayed presentation of tracheal injury is an exceptionally rare but significant complication following thyroidectomy that clinicians must be prepared to identify. A high index of clinical suspicion is necessary when a patient presents with a cough triggered by phonation or a cough-impulse neck swelling in the early postoperative weeks, even after an initially uneventful surgery. While the literature often emphasizes aggressive surgical repair for tracheal ruptures, this case demonstrates that small, stable, and contained defects can be successfully managed with a conservative, nonoperative approach. Maintaining vigilance during the first two weeks of follow-up is essential to ensure the prompt diagnosis and favorable management of such unusual airway complications, ultimately avoiding unnecessary invasive interventions in stable patients.
